# Overhydroxylation of Lysine of Collagen Increases Uterine Fibroids Proliferation: Roles of Lysyl Hydroxylases, Lysyl Oxidases, and Matrix Metalloproteinases

**DOI:** 10.1155/2017/5316845

**Published:** 2017-09-10

**Authors:** Marwa Kamel, Mohamed Wagih, Gokhan S. Kilic, Concepcion R. Diaz-Arrastia, Mohamed A. Baraka, Salama A. Salama

**Affiliations:** ^1^Department of Tumor Biology, Unit of Pharmacology, National Cancer Institute, Cairo University, Giza, Egypt; ^2^Department of Pathology, Faculty of Medicine, University of Beni Suef, Beni Suef, Egypt; ^3^Department of Obstetrics & Gynecology, University of Texas Medical Branch, Galveston, TX, USA; ^4^Department of Obstetrics, Gynecology and Reproductive Sciences, McGovern Medical School, Houston, TX, USA; ^5^Department of Clinical Pharmacy, Faculty of Pharmacy, Al-Azhar University, Cairo, Egypt; ^6^Department of Pharmacy Practice, College of Clinical Pharmacy, University of Dammam, Dammam, Saudi Arabia; ^7^Department of Pharmacology & Toxicology, Al-Azhar University, Cairo, Egypt

## Abstract

The role of the extracellular matrix (ECM) in uterine fibroids (UF) has recently been appreciated. Overhydroxylation of lysine residues and the subsequent formation of hydroxylysylpyridinoline (HP) and lysylpyridinoline (LP) cross-links underlie the ECM stiffness and profoundly affect tumor progression. The aim of the current study was to investigate the relationship between ECM of UF, collagen and collagen cross-linking enzymes [lysyl hydroxylases (LH) and lysyl oxidases (LOX)], and the development and progression of UF. Our results indicated that hydroxyl lysine (Hyl) and HP cross-links are significantly higher in UF compared to the normal myometrial tissues accompanied by increased expression of LH (LH2b) and LOX. Also, increased resistance to matrix metalloproteinases (MMP) proteolytic degradation activity was observed. Furthermore, the extent of collagen cross-links was positively correlated with the expression of myofibroblast marker (*α*-SMA), growth-promoting markers (PCNA; pERK1/2; FAK^pY397^; Ki-67; and Cyclin D1), and the size of UF. In conclusion, our study defines the role of overhydroxylation of collagen and collagen cross-linking enzymes in modulating UF cell proliferation, differentiation, and resistance to MMP. These effects can establish microenvironment conducive for UF progression and thus represent potential target treatment options of UF.

## 1. Introduction

Uterine fibroids (UF), also known as uterine leiomyomas, are benign smooth muscle neoplasms of the uterus found in almost 20–40% of women of reproductive age [1]. It is anticipated that up to 77% of women will develop UF in their life and 15 to 30% of these women suffer from substantial symptoms, including pelvic discomfort, menorrhagia, dysmenorrhea, anemia, urinary incontinence, recurrent pregnancy loss, preterm labor, and sometimes infertility [2]. The annual public cost for fibroids is estimated to be up to 34 billion dollars, calculated through combined expenses for management of symptomatic fibroids, diagnosis, and dealing with obstetrical complications of fibroids [3]. Consequently, finding effective therapeutic options is crucial for overcoming this major public health problem. An important step towards this goal is to explore the molecular basis of fibroids to understand and target the underlying specific pathophysiological pathways. Moreover, future research can identify potential targets which may help the development of new nonsurgical noninvasive treatments.

The uterine myometrium consists mostly of smooth muscle cells that stain positive for *α*-smooth muscle actin and desmin and are interspersed with interstitial collagens. The majority of collagens in the interstitial areas of the myometrium are Type I, Type III, and Type V, though types IV and VI are present in the uterus as well [4]. Type IV collagen is a membrane collagen, arrayed as a meshwork [5]. All collagens have three polypeptide chains and have repeated Gly-Xaa-Yaa sequences, where X is proline and Y is hydroxyproline. These peptide chains are able to accumulate into stable triple helical structures. Glycine is the smallest amino acid and its position in the collagen molecule allows for a firmly coiled helix as glycine is easily packed into the center of the assembled helix, while the hydroxyprolines are located on the outside of the triple helix stabilizing the molecule. Synthesis of collagen is in some way complicated. This involves the formation of pro-alpha chains, preprocollagen, procollagen, and tropocollagen fibrils and finally in the extracellular space copper-dependent lysyl oxidases (LOX) enzymatically create the intramolecular and intermolecular cross-links that form the mature collagen fibrils. These covalent cross-links are di-, tri-, and tetrafunctional and further stabilize the collagen helix [6]. The more lysine derived cross-links in collagenous tissue are, the stiffer is the tissue [7].

The extracellular matrix (ECM) comprises much of the UF mass which can be greater than 50% of the tumor size [8]. ECM, with its vital components and biophysical characteristics, represents the pathological microenvironment in which fibroid cells may develop. It was previously reported that ECM of UF is abnormally stiff which imposes increased mechanical stress on fibroid cells [9]. Indeed, substantial evidence suggests that the growth and morbidity associated with UF are related to excessive production and deposition of stiff, disorganized, and exceptionally stable ECM [10]. ECM acts as a reservoir for cytokines and growth factors and regulates their distribution, activation, and their presentation to cells [11]. Furthermore, ECM proteins act as solid phase ligands that can transduce and integrate complex multivalent signals and control cellular shape and architecture [12]. It is now well recognized that mechanotransduction affects cell behavior in fibrotic tissues through converting physical forces into biochemical signals thereby activating cellular receptors and stimulating intracellular pathways. Accordingly, cells that are exposed to the mechanical forces of collagen-rich fibrotic tissues are known to secrete more collagen and other components of the ECM and develop resistance to apoptosis leading to the persistence of cells [7].

The stiffness of fibroids ECM regulates cell proliferation and differentiation, collagen synthesis, and ECM remodeling. The stiff ECM can establish a microenvironment favorable for UF to reach a progressive and self-sustaining phase. [13]. Moreover, recent studies indicate that loss of stiffness may decrease bulk symptoms and possibly lead to shrinkage of fibroids through changed mechanotransduction leading ultimately to reduced fibroid symptoms of pain and bleeding [14]. There is ample evidence to suggest that stiffness of ECM is a result of overhydroxylation of lysyl residues and subsequent formation of cross-linked collagen molecules by the concerted action of lysyl hydroxylases (LH) [15, 16] and LOX [17]. These collagen cross-links are essential for assembling collagen molecules into the functional unit, deposition, and hardening of insoluble collagen fibers [18] and reducing the susceptibility of collagen to proteolytic degradation [19]. These findings raise the interesting possibility that overhydroxylation of lysyl residues and the subsequent formation of hydroxylysylpyridinoline (HP) and lysylpyridinoline (LP) collagen cross-links may be the underlying causes of increased stiffness of UF.

From all of the above, it can be understood that ECM plays important role in the development and progression of UF and that collagen cross-linking may have a fundamental part in this aspect. To address this, we thus investigated the biological relationship between collagen cross-linking and the development of UF and the role played by collagen cross-linking enzymes in this respect. No doubts that clarification of these issues is important, as they may provide novel drug targets for the prevention and/or treatment of UF.

## 2. Materials and Methods

### 2.1. Tissue Acquisition and Patient Background

The UF and adjacent normal appearing myometrial tissue samples were collected from premenopausal women (*n* = 20) who were scheduled to undergo hysterectomy due to symptomatic UF. The tissue specimens were obtained from the hospitals of the University of Texas Medical Branch (Galveston, TX). All specimens were collected after obtaining informed consent from subjects following protocols approved by the Institutional Review Board for Human Research. These women were not taking any medications containing GnRH analogs, oral contraceptives, or progestin for the previous three months before surgery. Based on their last menstrual periods and endometrial histology, they were from early-mid secretory phase of the menstrual cycle. All the fibroids used in this study were three to five cm in diameter and were collected one cm from the outer capsule. The adjacent myometrial tissues were sampled at two cm distance from the fibroid tumors. Immediately after sampling, portions of the tissues were frozen and kept in liquid nitrogen for further analysis (Table 1).

### 2.2. Measurement of Hydroxyproline

The UF and myometrial tissues stored in liquid nitrogen were washed thoroughly with PBS containing protease inhibitors, and the tissues were weighed and used for extraction. The hydroxyproline concentration was measured according to the method described by Lareu et al. [20]. Briefly, the tissue samples were air-dried and weighed and 100 mg of the tissues was subjected to acid hydrolysis in 6 N HCl for 16 hours at 110°C. The hydrolysate was suspended in an equal volume of 6 N NaOH and then filtered. The concentration of hydroxyproline was determined according to Woessner Jr.'s method [21].

### 2.3. Collagen Assay

Total collagen contents, soluble and insoluble (cross-linked collagens), were determined in UF and the normal myometrial tissues. Briefly, aliquots of tissue extracts (50 *μ*l) were used to determine collagen contents and the extent of collagen cross-linking using two colorimetric assays: Fast Green-Sirius Red to obtain total collagen [22] and Sircol-based assay to obtain soluble collagen [23]. The degree of collagen cross-linking was calculated as the ratio between the insoluble and the soluble forms of collagen.

### 2.4. Analysis of Collagen Cross-Links

Isolated ECM from the UF and corresponding normal myometrial tissue samples were hydrolyzed in HCl (6 N) and used for amino acid and cross-link analysis. The number of hydroxyl lysine (Hyl) residues was determined in the acid hydrolysates by reverse-phase high-performance liquid chromatography (RP-HPLC) after derivatization with 9-fluorenylmethyl chloroformate (FMOC) [24]. In the same hydrolysates, the number of the trifunctional Hyl-derived cross-links HP and LP was measured by HPLC as described previously [25, 26]. The number of pyridinoline cross-links and Hyl residues was expressed per collagen molecule assuming 300 hydroxyproline residues per triple helix.

### 2.5. Collagen Degradation

MMP-mediated collagen degradation was performed as previously described [27]. Briefly, fresh tissue samples (100 mg) were washed three times for 1 hour with serum-free culture medium. The tissues were sliced into small slices and incubated with recombinant active catalytic domain of MMP-2 (100 ng/ml) (Cat# BML-SE244-0010) or MMP-9 (100 ng/ml) (Cat#BML-SE237-0010, Enzo Life Sciences) and collagen degradation was monitored as the release of hydroxyproline. Hydroxyproline levels were determined using the colorimetric assay described by Creemers et al., 1997 [28].

### 2.6. Gene Expression Analysis

Total RNA was isolated from the tissue samples using the RNeasy kit (Qiagen). After that, mRNA was reverse-transcribed into cDNA using the oligo-dT primer (Roche). Quantitative real-time RT-PCR was then carried out using Applied Biosystems 7500 real-time RT-PCR system (Applied Biosystems, Foster City, CA) using TaqMan gene expression assays (Applied Biosystems) for LH1 (assay ID Hs00609368_m1), LH2 (assay ID Hs00168688_m1), LH3 (assay ID Hs01126612_m1), LOX (assay ID Hs00184700_m1), and glyceraldehyde-3-phosphate dehydrogenase (GAPDH) (assay ID Hs99999905_m1). The amount of RT-PCR product for the gene of interest was normalized to the quantity of GAPDH. Each assay was performed in triplicate.

### 2.7. Determination of LH2 Different Isoforms

To investigate the expression of the splicing variants of LH2 in UF and normal myometrial tissues, cDNA from UF and normal myometrial tissues was subjected to LH2a and LH2b PCR amplification. LH2a (145 bp) and LH2b (208 bp) were coamplified using specific forward (5′-TTAAAGGAAAGACACTCCGATCAGAGATGA-3′) and reverse primers (5′-TAGCCTTCCAAATTCATGTCTATTAGAAATGTA-3′) in a total reaction volume of 25 *μ*l containing 1x PCR buffer (Applied Biosystems), 0.4 mm each dNTP, 3.5 mm Mg^2+^, 500 nm each primer, and 1 unit of AmpliTaq Gold polymerase (Applied Biosystems). PCR was performed in a 9600 thermal cycler (PerkinElmer Life Sciences) and consisted of a 5-minute interval at 95°C followed by 35 cycles of 95°C for 30 seconds, 56°C for 40 seconds, and 72°C for 30 seconds. Aliquots of 15 *μ*l of each amplified sample were then subjected to electrophoresis in a 2% agarose gel containing ethidium bromide.

### 2.8. Western Blot Analysis

Tissue lysates were prepared from UF and normal myometrial tissues using mammalian protein extraction reagent supplemented with Halt protease inhibitor (Pierce, Rockford, IL, USA). Following tissue lysis, protein concentrations were determined by Bradford reagent using a Polar Star Optima microplate reader (BMG Labtechnologies, Durham, NC, USA). Equal amounts of protein (40 *μ*g) were then resolved by SDS-PAGE gel. The membranes were blocked with 5% nonfat milk in Tris-buffered Tween 20 buffer for 1.5 hours and then incubated overnight at 4°C with the corresponding primary antibodies. After three washes in Tris-buffered Tween 20 buffer, immunoreactive proteins were detected using horseradish peroxidase-conjugated secondary antibodies and enhanced chemiluminescence system (Amersham Pharmacia Biotech, Arlington Heights, IL). Bands were detected with X Omat film (Eastman Kodak Co., Rochester, NY). The radioautograms were scanned and quantified with Flurochem/*α* Ease FC imaging system (Alpha Innotech, San Leandro, CA). The relative intensities of each protein were normalized against the corresponding *β*-actin values.

### 2.9. Statistical Analysis

All data are expressed as means ± SEM. Differences between group means were detected by Student's *t*-test. Spearman's correlation coefficient test was used to examine the relationship between the level of collagen cross-links and the size of fibroid tumors, cell proliferation, and differentiation markers in UF. All analyses were performed by SPSS 11.5.0 for Windows. A two-tailed *P* value of less than 0.05 was considered significant.

## 3. Results

### 3.1. Collagen Content and Composition in UF and Normal Myometrial Tissues

**Figure 1:**
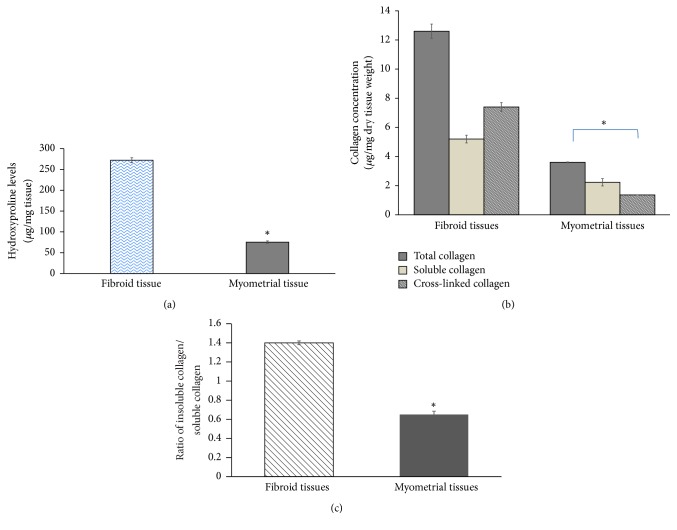
(a) Hydroxyproline levels, as a measure of total collagen, in fibroid tissues and normal myometrial tissues (*n* = 20): tissue samples were air-dried and weighed and 100 mg of the tissues was subjected to acid hydrolysis in 6 N HCl solution for 16 h at 110°C. The hydrolysates were suspended in an equal volume of 6 N NaOH and filtered. The concentration of hydroxyproline was determined according to Woessner's method. Hydroxyproline concentration was expressed as *μ*g per mg dry tissue weight. (b) The levels of total, soluble, and insoluble (cross-linked) collagen were determined in samples of UF and the adjacent normal myometrial tissues. Aliquots of tissue extracts (50 *μ*l) were used to determine the collagen contents and the extent of collagen cross-linking as described under* Materials and Methods*. Collagen concentration was expressed as *μ*g per mg dry tissue weight. (c) The ratio of insoluble:soluble forms of collagen in samples of UF and normal myometrial tissues. Values represent the means ± SEM (*n* = 20). ^*∗*^Significant difference at *P* < 0.05.

We started with the assessment of the total collagen content in the samples of UF and corresponding normal myometrial tissues (*n* = 20) by measuring the hydroxyproline content. As expected, our data indicated that total collagen is significantly higher in UF compared to the normal myometrial tissues. The mean values of hydroxyproline content in the UF and the normal myometrial tissues are 272.1 ± 11.8 and 75.3 ± 6.03 *μ*g/mg dry tissues, respectively (*P* < 0.05), (Figure 1(a)). Thus, the total collagen content in UF is 3.7-fold higher compared to the normal myometrial tissues.

Then, we studied whether there is an alteration in postsynthetic procollagen processing in UF compared to the normal myometrial tissues. Accordingly, we measured both insoluble and soluble collagen in UF and normal myometrial tissues. Our data clearly demonstrated that in UF tissues the increase in insoluble collagen content was more prominent than the increase in soluble collagen content. Thus, in UF, 41% of the collagen content was found to be soluble collagen, whereas 59% was insoluble collagen. In the normal myometrial tissues, the percentages of soluble and insoluble collagen were 62% and 38%, respectively (Figure 1(b)). Thus, the ratio of insoluble/soluble collagen was significantly higher in UF compared to the myometrial tissues. In the UF tissues, the insoluble/soluble collagen ratio was 1.4 ± 0.04, while in normal myometrial tissues, this ratio was 0.65 ± 0.09 (*P* < 0.01) (Figure 1(c)). This increase in insoluble/soluble collagen ratio in UF suggests a shift away from a relatively stable, easily degradable, less cross-linked, and soluble collagen to a relatively insoluble and highly cross-linked collagen which favors ECM deposition in UF.

### 3.2. Overhydroxylation of Lysine Residues and Increased Collagen Cross-Linking in Fibroid Tissues

We were interested in studying the hydroxylation patterns of lysine residues and their influence on collagen cross-linking in UF. Thus, we analyzed the amount of Hyl in UF and myometrial tissues. As indicated in Table 2, our data obviously showed overhydroxylation of lysine residues in collagen from UF compared to myometrial tissues. Thus, in UF the Hyl/collagen (mole/mole) is 57 ± 2.15 compared to 28 ± 3.4 in the myometrial tissues (*P* < 0.05). We then quantified HP and LP collagen cross-links in UF and myometrial tissues. Our data illustrated that the total amount of collagen cross-links (HP + LP) is significantly higher in UF compared to the myometrial tissues. As demonstrated in Table 2, the number of HP + LP/collagen (mmole/mole) in the UF is 201.1 ± 12.5 compared to 66.2 ± 2.3 in myometrial tissues (*P* < 0.05). Interestingly, relative abundance of HP and LP is considerably different in UF and myometrial tissues. In UF, the number of the HP collagen cross-links is significantly higher than the LP collagen cross-links. In contrast, the LP collagen cross-links are significantly higher than HP cross-links in the myometrial tissues. Another critical insight revealed by our study is that the numbers of HP cross-links per collagen molecules (mmole/mole) is significantly higher in UF tissues compared to the myometrial tissues (185.33 ± 13.7 versus 40.3 ± 3.7, resp., *P* < 0.05). In contrast, the number of LP cross-links/collagen molecule (mmole/mole) is significantly lower in UF compared to the normal myometrial tissues (15.7 ± 2.1 versus 25.9 ± 1.6, resp.; *P* < 0.05) (Table 2). The difference in the number of HP or LP collagen cross-links in UF and normal myometrial tissues is also reflected in the HP/LP ratio, which is significantly higher in UF compared with myometrial tissues (12.3 ± 2.4 versus 1.6 ± 0.22), respectively.

### 3.3. Increased Collagen Cross-Linking Is Associated with Increased UF Size

To assess the impact of collagen cross-linking on the growth of the UF, we correlated the level of collagen cross-links with UF size. Our data demonstrated that the levels of Hyl, HP, and LP positively correlate with UF size. The numbers of Hyl/collagen (mole/mole) in small (<20 mm), medium (20–50 mm), and large (>50 mm) UF are 36.4 ± 2.1; 56 ± 2.4; and 74 ± 3.4, respectively (*r* = 0.59; *P* = 0.006) (Table 3). Similarly, the numbers of HP/collagen (mmole/mole) are 121.4 ± 8.9; 157 ± 8.3; and 278 ± 9.7, respectively (*r* = 0.58; *P* = 0.007). Likewise, numbers of LP/collagen (mmole/mole) are 14.1 ± 1.2, 15.2 ± 1.3, and 20.3 ± 1.5, respectively (*r* = 0.51; *P* = 0.02). Consequently, the total of collagen cross-links (LP + HP) increased progressively as the size of fibroid tumor increased (*r* = 0.61, *P* = 0.006) (Table 3). These data suggest that elevated collagen cross-links, which reflect increased ECM stiffness, are associated with increased UF growth.

### 3.4. Increased Collagen Cross-Linking Enhances the Expression of Cell Proliferation Markers

**Figure 2:**
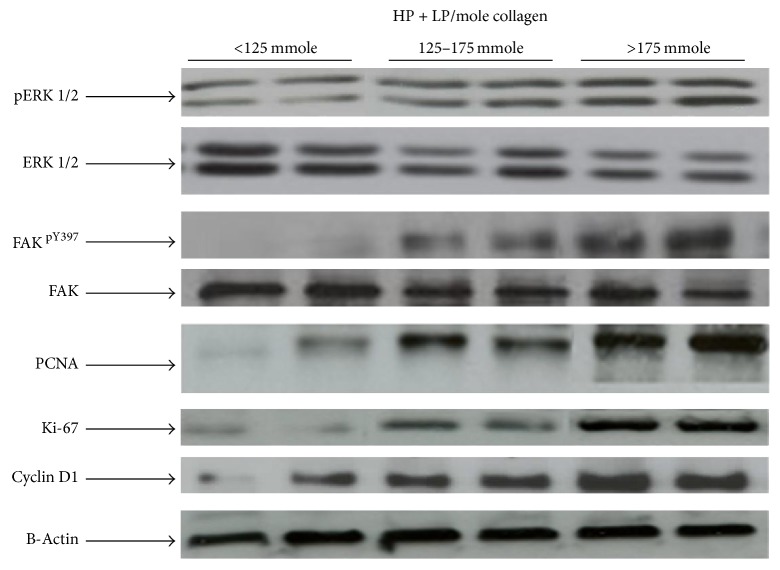
A representative immunoblotting showing the expression of FAK, ERK1/2, PCNA, Ki-67, and Cyclin D1 in UF tissues with different collagen cross-linking levels. *β*-Actin was used as an internal control. Band intensities of Western blots were quantified using an Alpha Imager System and corrected for *β*-actin.

We then investigated whether increased collagen cross-linking plays a role in the growth-promoting pathways in UF. Our data clearly demonstrated that the expression levels of PCNA; pERK1/2; FAK^pY397^; Ki-67; and Cyclin D1 are markedly increased as the collagen cross-linking increased (Figure 2). These results suggest that collagen cross-linking is a positive regulator of the cell cycle/cell proliferation pathways in UF.

### 3.5. Collagen Cross-Linking Is Associated with Myofibroblast Phenotype Expression in UF

**Figure 3:**
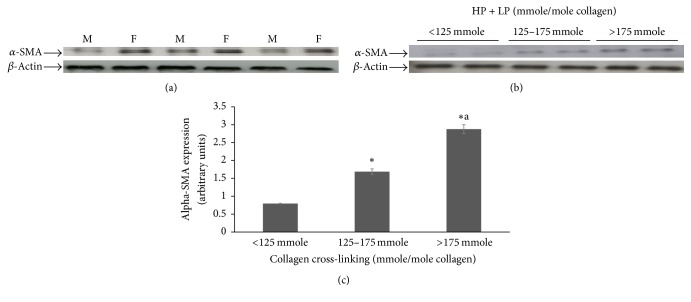
Expression of *α*-SMA in UF (F) and the adjacent normal myometrial tissues (M) and the effect of the extent of collagen cross-linking on *α*-SMA expression in fibroid tissues. (a) A representative immunoblotting of *α*-SMA expression in fibroids and normal myometrial tissues. *β*-Actin was used as an internal control. (b) A representative immunoblotting of *α*-SMA expression in UF tissues with different levels of collagen cross-linking. *β*-Actin was used as an internal control. (c) Band intensities of Western blots were quantified using an AlphaImager System and corrected for *β*-actin. *∗* represents significant difference from the <125 mmole group and *∗*a represents significant difference of 125–175 mmole group.

The next step was to understand the dynamic relationship and correlation between the levels of collagen cross-linking and the myofibroblast phenotype in UF by investigating *α*-smooth muscle actin (*α*-SMA) expression in UF and normal myometrial tissues. We noted an increase in *α*-SMA expression in UF compared to normal myometrial tissues (Figure 3(a)). In addition, our data revealed that the extent of *α*-SMA expression positively correlates with the level of collagen cross-linking (*r* = 0.63, *P* = 0.01) (Figures 3(b) and 3(c)). These data establish an association between increased collagen cross-linking and the expression of myofibroblast phenotype in UF tissues.

### 3.6. Collagen Cross-Linking Determines the Expression and Activities of MMP in UF

**Figure 4:**
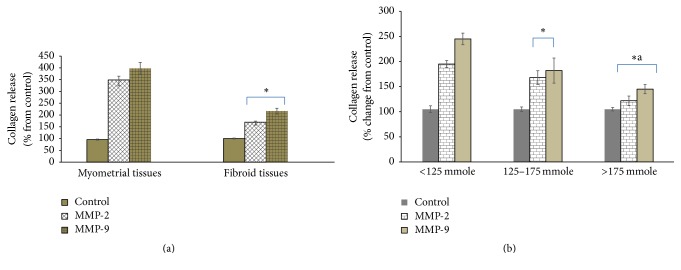
Comparison of the effect of exogenous, purified, and active MMP-2 or MMP-9 on collagen release from UF and normal myometrial tissue slices (a) or UF tissues expressing different levels of collagen cross-linking (b). Tissue slices (100 mg) of UF and the normal myometrial tissues were incubated with purified and active MMP-2 or MMP-9 (100 ng/ml for 48 h at 37°C), and the released hydroxyproline was determined as a measure of collagen degradation. The released collagen was calculated as % of corresponding untreated control tissues. Values represent the means ± SEM. *∗* indicates a significant difference from control myometrium group (*P* < 0.05) (a) or *∗* indicates a significant difference from the <125 mmole group (*P* < 0.05) and *∗*a indicates a significant difference from the 125–175 mmole group (*P* < 0.05) (b).

To verify whether increased collagen cross-linking in UF confers resistance to the proteolytic effects of MMP-2 and MMP-9, exogenous, purified, and active recombinant MMP-2 or MMP-9 was added to UF or myometrial tissue slices followed by measuring collagen release. Addition of 100 ng/ml of MMP-2 or MMP-9 to cultured slices of myometrial tissue slices caused an increase in the collagen release by 2.6-fold and 3.1-fold, respectively, compared with untreated control myometrial tissue slices (Figure 4(a)). On the contrary, MMP-2 or MMP-9 (100 ng/ml) increased the collagen release only by 0.7-fold and 1.2-fold, respectively, compared with the untreated control UF tissue slices (Figure 4(a)). Based on these results, we conclude that the collagen in UF is less susceptible to the proteolytic effect of MMP as a consequence of increased levels of collagen cross-linking. Furthermore, we assessed whether the magnitude of proteolytic effect of MMP-2 and MMP-9 on UF tissues correlates with the extent of collagen cross-links. As indicated in (Figure 4(b)), increased collagen cross-linking is associated with increased inhibition of the proteolytic effect of MMP-2 and MMP-9 on UF tissues.

### 3.7. Expression of Collagen Cross-Linking Enzymes in UF Coincides with Increased Collagen Cross-Linking

**Figure 5:**
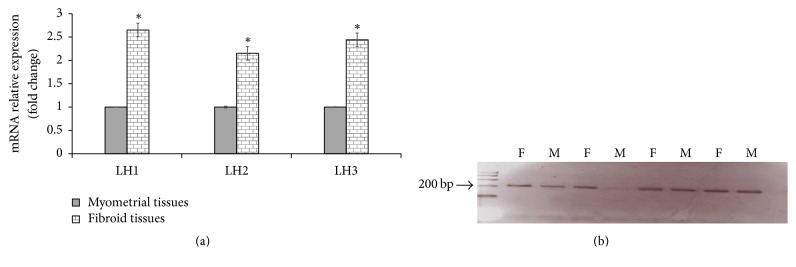
(a) Expression of three different isoforms of LH1, LH2, and LH3. The mRNA levels in samples of UF (F) and adjacent myometrial tissues (M) (*n* = 20) were measured by quantitative real-time RT-PCR and were normalized to GAPDH mRNA levels. All values in fibroid tissues were described as fold differences compared to those measured in the corresponding myometrial tissues. Results are presented as mean ± SD. *∗* indicates a significant difference from control myometrium (*P* < 0.05). (b) PCR amplification of different isoforms of LH2: cDNA from samples of UF and normal myometrial tissues were PCR amplified using primers for LH2a and LH2b. Aliquots of each amplified sample were subjected to electrophoresis in a 2% agarose gel containing ethidium bromide. The expected band sizes were 145 bp for LH2a and 208 bp for LH2b.

**Figure 6:**
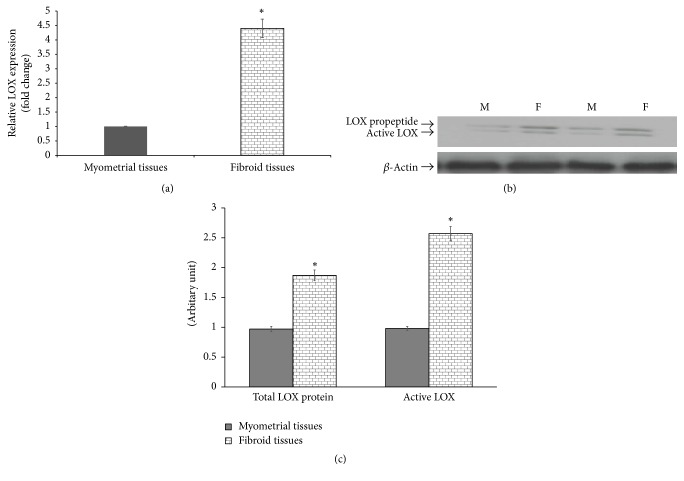
Expression of LOX gene in UF and normal myometrial tissues. (a) Quantitative real-time RT-PCR analysis shows a significant increase in the expression of LOX gene in fibroid tissues compared with the normal myometrial tissues (*n* = 20). (b) Expression of pro-LOX and active LOX proteins in fibroids and normal myometrial tissues. (c) Quantification of the total and active LOX proteins in fibroids and normal myometrial tissues. The bands corresponding to the total and active LOX proteins were analyzed by densitometric scan, using *β*-actin for normalization. *∗* indicates a significant difference (*P* < 0.05).

To determine the extent of the involvement of collagen cross-linking enzymes LH and LOX in UF, we investigated the expression of the three different isoforms of LH (LH1, LH2, and LH3) and LOX genes in UF and normal myometrial tissues. Our data revealed that UF express higher level of LH1, LH2, and LH3 compared to the normal myometrial tissues (Figure 5). These data clearly demonstrate that LH1–3 expression appears to be of significant importance in UF development. Interestingly, LH2 is exclusively expressed in both UF and normal myometrial as LH2b. Collectively, these data suggest that the expression level rather than the pattern of splicing isoforms of LH2 gene may contribute to UF development. Regarding LOX expression, the quantitative real-time RT-PCR analysis reveals that LOX expression is higher at the mRNA level in UF compared to normal myometrial tissues (Figure 6(a)). It was interesting to investigate whether pro-LOX or the active LOX protein is differentially expressed in UF and myometrial tissues. Our data showed that the amount of both pro-LOX (50 kDa) and active LOX (32 kDa) is remarkably higher in UF compared to the normal myometrial tissues (Figures 6(b) and 6(c)). Together, our data demonstrate that increased expression of LH and LOX coincides with increased collagen cross-linking and suggests that overexpression of LH and LOX might play a significant role in the development of UF.

## 4. Discussion

Fibroids are benign smooth muscle neoplasms of the uterus and the most common pelvic tumors in women of reproductive age. They are the leading cause of hysterectomies worldwide and the most common indication for gynecological surgeries in the US. UF are found in almost 20–40% of women of reproductive age [1, 29].

The cause of uterine leiomyomas remains unknown. Estrogen and progesterone are considered the most important regulators of leiomyoma growth but the current understanding is that stem cells, genetic and epigenetic factors, sex steroids, growth factors, cytokines, chemokines, and ECM components are important factors involved in the development and growth of leiomyomas [30–35].

ECM is an important factor of leiomyoma growth, and it may serve as a reservoir for growth factors, cytokines, chemokines, and angiogenic and inflammatory response mediators [36–38]. The ECM of UF consists mainly of Types I and III collagens and represents the majority of the fibroid weight [10]. One hallmark of ECM of UF is its increased stiffness. Like many other tumors and fibrotic diseases, stiffness of ECM may enrich the tumor growth. Indeed, previous studies have reported that the stiffness of the ECM of fibroids imposes increased mechanical stress on the fibroid cells [9]. In addition, this stiffness can alter the effectiveness of therapeutic agents by slowing down the movement of drug molecules within the tumor [39]. Definitely, UF should be regarded not only as dysregulated cellular signaling and behavior, but also as a context-dependent pathology where fibrotic cells interact with and respond to the rigidity of ECM. Despite the fact that the biophysical characteristics and the nature of UF ECM are of both scientific and clinical importance, the molecular mechanisms underlying the abnormal stiffness of ECM and its biological consequences remain poorly understood in this highly prevalent disease. In order to address these and related issues, we investigated the collagen cross-link formation and the expression of collagen cross-linking enzymes LH1–3 and LOX in collected samples of UF and normal myometrial tissues. Overall, the study's novelty lies in uncovering some mechanisms underlying the accumulation of ECM in UF and suggesting potential therapeutic targets for this disease. The main original findings of this study are as follows: (i) lysine residues are overhydroxylated in UF compared to normal myometrial tissues; (ii) collagen in UF is mainly cross-linked insoluble collagen; (iii) both mature collagen HP and LP cross-links are present in UF but HP is present at a higher level, whereas in normal myometrial tissues, the LP collagen cross-links are higher than HP; (iv) collagen cross-linking in UF is associated with resistance of ECM to the degrading effects of MMP-2 and MMP-9; (v) collagen cross-linking enzymes LH1–3 and LOX are highly expressed in UF compared to the normal myometrial tissues.

Overhydroxylation of lysine residues and increased level of Hyl-derived collagen cross-linking in UF demonstrated by our study can be understood in the light of the importance of posttranslational modifications (e.g., degrees of lysine hydroxylation) and preferred intermolecular binding partners for telopeptide and helical cross-linking domains in regulating cross-link type and placement [40]. These findings are also consistent with the previous reports that hydroxylation of specific lysine residues is one of such unique modifications found in collagen, and the pattern/extent of this modification influences fibrillogenesis, cross-linking, and ECM deposition [16]. Increased Hyl is thought to play a pivotal role in fibrosis by exerting two important functions, which are probably involved in the etiology of UF: (i) they are essential and prerequisite for the formation and stability of the intermolecular collagen cross-links, and consequently accumulation of ECM [41]; (ii) they serve as attachment sites for carbohydrate units to form advanced glycation end products (AGEs) [42], which has been documented to promote ECM deposition and expression of profibrotic cytokines in many fibrotic disorders [43]. Indeed, analysis of collagen cross-linking confirmed that collagen cross-links are significantly higher in UF compared to the normal myometrial tissue. As mentioned above, our data demonstrated that collagen in UF exhibits a cross-linking pattern that is predominantly HP, while cross-linking present in normal myometrial tissues collagen is predominantly LP. Formation of HP or LP collagen cross-links depends on the hydroxylation status of the lysyl residues in the collagen telopeptides. Lysyl residues overhydroxylation, as observed in UF, favors the Hyl-aldehyde cross-linking pathway with subsequent formation of high levels of HP cross-links [44, 45]. Thus, it is conceivable that collagen cross-linking is a distinctive phenotype of UF collagen and may play a role in the development and maintenance of this disease. Consistent with our finding, previous studies reported that Hyl collagen cross-linking is a general fibrotic phenomenon which has consistently been found in several human fibrotic disorders, for example, skin fibrosis [46].

The extent of Hyl-derived collagen cross-links was found to be related to the irreversible accumulation of collagen in injured skin [47] and liver fibrosis [19, 48]. This led us to hypothesize that their presence may decrease the degradability of the UF. Indeed, our results clearly indicated that collagen in UF is resistant to MMP-induced degradation. This finding is in agreement with previous findings that collagen cross-linking confers collagen resistance to the natural turnover processes [18] and reduces its susceptibility to MMP-induced proteolytic degradation [49, 50]. Indeed, previous studies indicated that UF express higher levels of MMP-2 and MMP-9 compared to normal myometrial tissues [51–53]. The increased expression of MMP in UF is probably a result of positive compensation for the increased ECM accumulation due to collagen cross-linking. Resistance of collagens in UF to degradation with concomitant increase in MMP secretion and activation can contribute to UF growth. In addition, MMP are important key players in the process of ECM remodeling allowing tumor cells to modify the ECM and release cytokines, growth factors, and other cell-surface molecules, ultimately facilitating protease-dependent tumor progression [54].

We then explored the effect of collagen cross-linking on UF phenotype. Our data revealed that increased collagen cross-linking is associated with increased UF size as well as increased expression of cell proliferation and differentiation markers. These findings are consistent with previous findings that increased collagen cross-linking enhances ECM stiffness, which in turn is a positive regulator of the cell cycle through a highly selective effect on integrin-dependent signaling to FAK, Rac, and Cyclin D1 [55].

Our data indicated that the extent of collagen cross-linking was positively correlated with the expression of the myofibroblast marker *α*-SMA. It is well known that fibroblasts play a central role in building, maintaining, and remodeling of ECM. Under physiological conditions, fibroblasts produce collagens, elastins and glycosaminoglycans, and ECM-modulating proteases like MMPs. Under fibrotic conditions, fibroblasts transdifferentiate into myofibroblasts mainly induced by TGF-*β*. Activated myofibroblasts contribute to disease progression by excessive production of ECM with collagen I being the major component. Moreover, myofibroblasts are characterized by elevated expression of *α*-SMA resulting in stress fiber formation and increased contractibility [56]. Notably, high levels of *α*-SMA were considered to be negatively associated with idiopathic pulmonary fibrosis patient survival [57]. Thus, our data suggest that the effect of collagen cross-linking on ECM stiffness and remodeling may have important clinical impact on UF progression by upregulating the expression of myofibroblast phenotype and activity. Once the myofibroblast phenotype gets expressed, it is apparently difficult to control and to terminate its harmful activity. Partly, this is caused by the constant mechanical feedback that the fibroid cells receive from the stiff ECM: stiffer ECM leads to higher myofibroblast contraction and ECM proteins secretion, which again leads to further ECM stiffening, and vicious cycles will be set in motion. Thus, it is therapeutically rational to interrupt the myofibroblast phenotype expression—ECM stiffening loop—by inhibiting collagen cross-linking and consequently the ECM stiffness [58]. Inherent questions remain open on what starts the mechanical feedback loop with the ECM; are fibroblasts first becoming contractile to stiffen the ECM or is ECM stiffness preceding the fibrotic process? Although our findings cannot precisely delineate whether ECM stiffness is a cause or a sequence, it has been suggested that ECM stiffness precedes the fibrotic process and determines the cell behavior [59].

To further understand the reasons behind the increased collagen cross-linking and ECM stiffness in UF, we evaluated the expression of collagen cross-linking enzymes LH and LOX in UF and normal myometrial tissues. The LH family consists of three lysyl hydroxylase isoforms (LH1, LH2, and LH3) [60, 61]. Our data demonstrated that different isoforms of LH and LOX are highly expressed in UF compared to the normal myometrial tissues. Among the different LH isoforms which are highly expressed in UF is the alternatively spliced LH2 enzyme, which acts as a collagen telopeptide hydroxylase. Our study clearly demonstrated that LH2 is exclusively expressed as LH2b isoform in UF. However, normal myometrial tissues also express LH2b splicing variant. Thus, the quantitative difference in LH2b rather than expression of different splicing variants could be the reason underlying the increased collagen cross-links and ECM. No doubts that increased expression of LH2b is linked to fibrotic diseases [62]. Similarly, we found that LH3 is highly expressed in UF. LH3 has three unique posttranslational modifications present in collagenous sequences in vivo and it hydroxylates lysyl residues, galactosylates hydroxylysyl residues, and glucosylates galactosyl hydroxylysyl residues [63].

Concerning the lysyl oxidase family, it consists of five members in mammals designated LOX and lysyl oxidase like-1 through lysyl oxidase like-4 (LOXL1–LOXL4). LOX is required for the biosynthesis of normal collagens and elastin, and therefore LOX is critical for vascular, mineralized, and nonmineralized connective tissues. In fibrotic diseases, elevated LOX activity is consistently observed and contributes to resistance of the extracellular matrix to proteolytic degradation, thus contributing to connective tissue accumulation and fibrosis. Data constantly show that LOX is upregulated in a variety of fibrotic conditions, sometimes accompanied by additional LOX isoforms, including lung, liver, heart, and skin fibrosis [64]. LOX mediates covalent cross-linking of collagen and the formation of insoluble collagen [65]. LOX is synthesized as an inactive precursor, pro-LOX, and then activated by proteolytic cleavage of the LOX propeptide [66]. Our results demonstrate that LOX expression is significantly higher in UF compared to myometrial tissues. In addition, UF demonstrate increased proteolytic activation of LOX. In addition to its effect on collagen, LOX interacts with fibronectin, another stromal ECM component that can modulate matrix stiffness [67]. Intriguingly, LOX action in cross-linking the ECM may oppose the role of matrix-degrading effects of MMP. LOX is likely to collaborate with MMP to generate a dynamic microenvironment that mechanically and chemically influences matrix remodeling. In harmony with our results, very recently it was demonstrated that lysyl oxidase family members especially LOX-like 2/3 play important roles in pulmonary fibrosis [68]. These findings together support the potential therapeutic value in targeting LOX for fibroid treatment. It is noteworthy that very significant progress in developing small molecule pan-lysyl oxidase isoform inhibitors and isoform-specific LOX inhibitors is a promising current avenue of ongoing research that seems likely to lead to new therapies for fibrosis and possibly metastatic cancer [64].

The increased expression of LH and LOX in UF can be explained on the light of the prior findings that several mediators such as TGF-*β*1 [69], connective tissue growth factor [70], bone morphogenetic protein-1 (BMP-1) [71], and reactive oxygen species [72] are highly expressed in UF, are well-known inducers for LH and LOX and are also inducers of proteolytic activation of LOX [73], and are consequently implicated in UF development.

Taken together, the current study pinpoints the role of collagen cross-linking and collagen cross-linking enzymes in UF and raises several possibilities that modulation of the collagen cross-linking/cross-linking enzymes could serve as potential targets for establishing a nonpermissive microenvironment that may prevent the progression or even induce remission of the UF. It will be exciting to see these findings being exploited therapeutically and used to develop new drugs that prevent or possibly even reverse established UF.

## Figures and Tables

**Table 1:** Characteristics of the study population.

Patient #	Age	Fibroid size (mm)	Location
(1)	35	2.4 × 3.2 × 2.5 = 16.8	Intramural
(2)	37	3.4 × 4.2 × 4.5 = 64.3	Intramural
(3)	35	3.5 × 2.9 × 3.2 = 32.5	Submucosal
(4)	37	3.5 × 4.5 × 3.7 = 58.3	Intramural
(5)	42	3.2 × 2.3 × 2.4 = 17.7	Submucosal
(6)	39	2.1 × 3.3 × 2.6 = 18.01	Submucosal
(7)	37	1.5 × 3.2 × 2.4 = 11.52	Intramural
(8)	37	2.2 × 3.3 × 1.6 = 11.2	Intramural
(9)	38	3.2 × 2.1 × 3.3 = 22.2	Submucosal
(10)	44	2.5 × 2.9 × 3.1 = 22.5	Intramural
(11)	42	6.8 × 8.3 × 6.9 = 389	Subserosal
(12)	39	3.2 × 4.3 × 2.5 = 34.4	Intramural
(13)	35	2.3 × 3.9 × 3.1 = 27.9	Intramural
(14)	38	5.7 × 6.9 × 4.8 = 189	Intramural
(15)	36	5.5 × 4 × 5 = 110	Submucosal
(16)	37	6.7 × 4.8 × 7.9 = 254	Subserosal
(17)	42	6.5 × 5.4 × 4.3 = 981	Intramural
(18)	44	3.4 × 3.6 × 2.1 = 25.7	Subserosal
(19)	35	6.3 × 5.8 × 4.9 = 179.1	Intramural
(20)	38	2.3 × 4.4 × 3.3 = 33.4	Subserosal

**Table 2:** Hyl residues and collagen cross-links in samples of UF and normal myometrial tissues. Hyl and collagen cross-links, HP, and LP were measured in samples (*n* = 20) of UF and normal myometrial tissues as described in *Materials and Methods*. The numbers of Hyl, HP, and LP were expressed per collagen molecule assuming 300 hydroxyproline residues per triple helix. Data are represented as means ± SEM (*n* = 20). ^*∗*^Significant difference compared to the normal myometrial tissues (*P* < 0.05).

	Hyl/collagen (mol/mol)	HP/collagen (mmol/mol)	LP/collagen (mmol/mol)	HP + LP/collagen (mmol/mol)	HP/LP (mol/mol)
Fibroid tissues	57 ± 2.15^*∗*^	185.33 ± 13.7^*∗*^	15.7 ± 2.1^*∗*^	201 ± 12.5^*∗*^	12.3 ± 2.4^*∗*^
Myometrial tissues	28 ± 3.4	40.3 ± 3.7	25.9 ± 1.6	66.2 ± 2.3	1.6 ± 0.22

**Table 3:** Hydroxylysine (Hyl), hydroxylysylpyridinoline (HP), and lysylpyridinoline (LP) levels in small, medium, and large UF and their matched myometrial tissues. Hyl, HP, and LP were measured by HPLC. The numbers of Hyl, HP, and LP were expressed per collagen molecule assuming 300 hydroxyproline residues per triple helix. Data are represented as means ± SEM. ^a^Significant difference compared to the small fibroids (*P* < 0.05). ^b^Significant difference compared to the small and medium fibroids (*P* < 0.05).

	Small (<20 mm; *n* = 5)		Medium (20–50 mm; *n* = 7)		Large (>50 mm; *n* = 8)	
	Fibroids	Myometrium	Fibroids	Myometrium	Fibroids	Myometrium
Hyl/collagen (mole/mole)	36.4 ± 2.1	31.2 ± 1.9	56 ± 2.4^a^	26.5 ± 1.6	74 ± 3.4^b^	27.4 ± 2.1
HP/collagen (mmole/mole)	121 ± 8.9	48 ± 3.1	157 ± 8.3^a^	32 ± 2.9	278 ± 9.7^b^	38 ± 3.2
LP/collagen (mmole/mole)	14.1 ± 1.2	28.1 ± 2.1	15.2 ± 1.3	23.5 ± 2.4	20.3 ± 1.5^b^	23.4 ± 1.8
HP+LP/collagen (mmole/mole)	135.1 ± 6.1	76.1 ± 2.8	172.2 ± 7.9^a^	55.5 ± 4.2	298.3 ± 5.9^b^	61.4 ± 2.1
HP/LP (mmole/mmole)	8.9 ± 0.45	1.7 ± 0.02	10.3 ± 0.1	1.4 ± 0.05	13.9 ± 1.2^b^	1.6 ± 0.03
