# Erratum to “Overhydroxylation of Lysine of Collagen Increases Uterine Fibroids Proliferation: Roles of Lysyl Hydroxylases, Lysyl Oxidases, and Matrix Metalloproteinases”

**DOI:** 10.1155/2018/2420836

**Published:** 2018-07-18

**Authors:** Marwa Kamel, Mohamed Wagih, Gokhan S. Kilic, Concepcion R. Diaz-Arrastia, Mohamed A. Baraka, Salama A. Salama

**Affiliations:** ^1^Department of Tumor Biology, Unit of Pharmacology, National Cancer Institute, Cairo University, Giza, Egypt; ^2^Department of Pathology, Faculty of Medicine, University of Beni-Suef, Beni-Suef, Egypt; ^3^Department of Obstetrics & Gynecology, University of Texas Medical Branch, Galveston, Tx, Usa; ^4^Department of Obstetrics, Gynecology and Reproductive Sciences, Mcgovern Medical School, Houston, Tx, Usa; ^5^Department of Clinical Pharmacy, Faculty of Pharmacy, Al-Azhar University, Cairo, Egypt; ^6^Department of Pharmacy Practice, College of Clinical Pharmacy, University of Dammam, Dammam, Saudi Arabia; ^7^Department of Pharmacology & Toxicology, Al-Azhar University, Cairo, Egypt

In the article titled “Overhydroxylation of Lysine of Collagen Increases Uterine Fibroids Proliferation: Roles of Lysyl Hydroxylases, Lysyl Oxidases, and Matrix Metalloproteinases” [1], there was an error in the affiliation of the second author. The corrected affiliations are shown above.
